# Study protocol the Continuing Care Project: a randomised controlled trial of a continuing care telephone intervention following residential substance dependence treatment

**DOI:** 10.1186/s12889-020-8206-y

**Published:** 2020-01-28

**Authors:** Peter Kelly, Frank Deane, Gerard Byrne, Tayla Degan, Briony Osborne, Camilla Townsend, James McKay, Laura Robinson, Christopher Oldmeadow, Kenny Lawson, Andrew Searles, Joanne Lunn

**Affiliations:** 1 School of Psychology, University of Wollongong, Northfields Avenue, Wollongong, New South Wales 2522 Australia; 2 Illawarra Health and Medical Research Institute, University of Wollongong, Northfields Avenue, Wollongong, New South Wales 2522 Australia; 3 The Salvation Army, Chalmers Street, Redfern, New South Wales 2016 Australia; 4 University of Pennsylvania, Market Street, Philadelphia, PA 19104 USA; 5 Hunter Medical Research Institute, Kookaburra Circuit, New Lambton Heights, New South, Wales 2305 Australia; 6 We Help Ourselves, Rozelle, New South Wales, 2039 Australia

**Keywords:** Continuing care, Telephone, The salvation Army, We help ourselves, Substance use, Methamphetamine, Alcohol, Mental health

## Abstract

**Background:**

A priority area in the field of substance dependence treatment is reducing the rates of relapse. Previous research has demonstrated that telephone delivered continuing care interventions are both clinically and cost effective when delivered as a component of outpatient treatment. This protocol describes a NSW Health funded study that assesses the effectiveness of delivering a telephone delivered continuing care intervention for people leaving residential substance treatment in Australia.

**Methods/design:**

All participants will be attending residential alcohol and other drug treatment provided by The Salvation Army or We Help Ourselves. The study will be conducted as a randomised controlled trial, where participants will be randomised to one of three treatment arms. The treatment arms will be: (i) 12-session continuing care telephone intervention; (ii) 4-session continuing care telephone intervention, or (iii) continuing care plan only. Baseline assessment batteries and development of the participants’ continuing care plan will be completed prior to participants being randomised to a treatment condition. Research staff blind to the treatment condition will complete follow-up assessments with participants at 3-months and 6-months after they have been discharged from their residential service.

**Discussion:**

This study will provide comprehensive data on the effect of delivering the continuing care intervention for people exiting residential alcohol and other drug treatment. If shown to be effective, this intervention can be disseminated to improve the rates of relapse among people leaving residential alcohol and other drug treatment.

**Trial registration:**

Australian New Zealand Clinical Trials Registry, ACTRN12618001231235. Registered on 23rd July 2018. https://www.anzctr.org.au/Trial/Registration/TrialReview.aspx?id=375621&isReview=true

## Background

Relapse rates are high for people attending alcohol and other drug (AOD) treatment [1]. Studies indicate that between 40 to 70% of participants leaving residential services report some use of alcohol or other drugs in the first six months after leaving residential treatment [2, 3]. To improve treatment outcomes and reduce relapse rates, continuing care interventions are recommended [4–6].

‘Aftercare’ services provided by residential services are typically focused on referral only (e.g., referral to services in the person’s local area, referral to 12-step groups). It is likely that adding a more ‘active’ continuing care component to treatment as usual will help to reduce relapse rates and improve participants’ transition back into the community [4–6]. Several modalities for delivering active continuing care have been studied, these include cognitive behavioural therapy, motivational interviewing, 12-step oriented and process groups. There are now a number of reviews [5, 6] and meta-analyses [4] that support the use of continuing care to promote longer-term outcomes. Across a variety of treatment settings (e.g., following detoxification, outpatient treatment or residential programs) and using different forms of continuing care (e.g. face to face, attendance at mutual support groups) the effects are significant (g = 0.27, *p* < .01, *n* = 13) [4]. Despite likely benefits of continuing care interventions, implementation can be hampered by low uptake [7], lack of service resources [8], ease of access (e.g., to transport) [6] and individual characteristics of the person leaving treatment (e.g., beliefs and attitudes towards after-care, level of motivation, readiness to change) [8, 9].

Telephone based continuing care is likely to be a well-suited modality for overcoming several of these barriers. McKay and colleagues have developed a standardised Continuing care telephone intervention [10] that has been successfully trialled in the United States to support people who have completed intensive outpatient treatment [10]. In a randomised controlled trial, telephone delivered continuing care (i.e., 12-sessions, the first session was completed face-to-face and then subsequent sessions were completed over the telephone) was compared with other forms of more intensive continuing care. These included face-to-face relapse prevention training (1x individual session and 1 x group session per week) and treatment as usual (2 × 12-step groups per week for 12-weeks). Results demonstrated that the telephone intervention was as effective as the more intensive approaches to continuing care in increasing the percentage of days abstinent and reducing negative consequences of substance use. Encouragingly, the participants completing the telephone condition demonstrated a higher percentage of days abstinent than the standard condition.

Subsequent economic analysis of the telephone approach has found that it is cost effective to deliver [6, 10–12]. The protocol developed by McKay is listed on SAMHSA’s National Registry of Evidence Based Programs and Practices (NREPP). However, published research has not examined the effectiveness of this program to support people leaving therapeutic communities, nor has it examined the intervention within an Australian context.

### Objectives

The Continuing Care Project will examine the continuing care intervention developed by McKay and colleagues (2005) for people exiting residential AOD treatment in Australia. The proposed study addresses calls in the broader academic literature to conduct well-controlled studies in this field [4–6]. It is hypothesised that: (i) participants in the continuing care treatment arms will demonstrate significantly higher percentages of days abstinent from alcohol and other drugs (excluding tobacco) at follow-up compared to the control (continuing care plan only) arm; (ii) that participants in the 12-session continuing care arm will demonstrate higher percentages of days abstinent at follow-up compared to the 4-session arm. Study results will also provide important information on the cost effectiveness of including continuing care telephone interventions as part of routine rehabilitation services.

## Methods

### Setting

Participants will be attending residential AOD treatment provided by The Australian Salvation Army and We Help Ourselves (WHOS). All of the treatment sites were located in New South Wales, Australia. The Salvation Army programs are: William Booth House (102 beds, including 82 for males and 20 for females) and the Dooralong Transformation Centre (150 beds, including 110 for males and 40 for females). The treatment program across both sites is a minimum 3 months in length and is operated in the form of a modified therapeutic community. Previous research has described these programs and examined the characteristics of people accessing these services [13–17]. The WHOS sites are: Gunyah (29 male beds) and New Beginnings (19 female beds). WHOS is operated in the form of a therapeutic community and participants may stay for 3 to 4 months. Previous research has also described the WHOS program characteristics or participants attending these programs [18, 19].

### Design

**Fig. 1:**
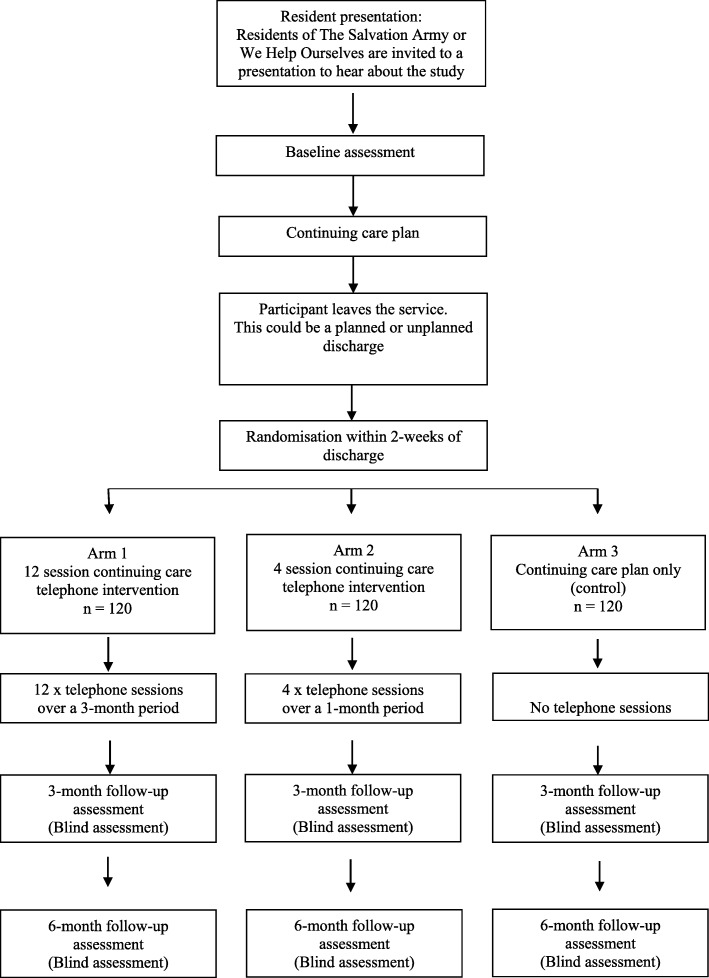
Study flow chart

A multi-centre prospective, randomised, open, blinded endpoint (PROBE) design will be utilised to compare the three study conditions. All three study arms will include usual care (i.e. aftercare planning, referral to 12-step meetings) and the completion of a continuing care plan. The study arms are as follows; (i) 12-session continuing care telephone intervention over a 3-month period following discharge from the treatment program; (ii) 4-session continuing care telephone intervention over a 1-month period, following discharge from the treatment program, or (iii) usual care plus a continuing care plan only. Assessments will be conducted at baseline, at 3-months post discharge from the treatment program, and at 6-months post discharge. At each assessment time point the assessment officers will be blind to the treatment condition. Fig. 1 describes the study flow.

The protocol follows Standard Protocol Items: Recommendations for Interventional Trials (SPIRIT) guidelines (see Table 1 and Additional File 1 SPIRIT checklist).

**Table 1:** SPIRIT table

	Study Period					
	Recruitment	Baseline	Randomisation	Intervention	Follow-up	
Week	0	0	^a^	1–12	3-month	6-month
Contacted by	CCW	CCW	CCW	CCW	RA	RA
ENROLMENT						
Eligibility screen	X					
Informed consent	X					
Baseline assessment		X				
Continuing Care Plan		X				
Allocation			X			
INTERVENTION						
Continuing Care Project × 12 sessions				~Week 1–12	X	X
Continuing Care Project × 4 sessions				~Week 1–4	X	X
Treatment as Usual				–	X	X
ASSESSMENTS						
Demographic information		X				
Timeline Follow Back		X			X	X
Substance Use Recovery Evaluator (SURE)		X			X	X
Drug Taking Confidence Questionnaire (DTCQ-8)		X			X	X
Lifetime Drug Use History (LDUH; Sections 11 and 12)		X			X	X
Desires for Alcohol Questionnaire (DAQ-6)						
EUROHIS QOL 8-item index		X			X	X
Kessler-10 (K10)		X			X	X
Short-Form-12 (SF-12)		X			X	X
Brief Treatment Outcome Measure: Blood Borne Virus Risk items		X			X	X
Heaviness of Smoking Index (HIS)		X			X	X
UCLA Loneliness measure (3-items)						
Health Literacy Questionnaire (HLQ)		X			X	X
Eating Disorder Screen for Primary Care (ESP)		X			X	X
Client Services Receipt Inventory (CSRI)		X			X	X

^a^randomization occurs when the participant leaves the service – which could be planned or unplanned discharge. *CCW* Continuing Care Worker, *RA* Research Assistant blind to treatment allocation

### Participants

A total of 360 people will be recruited to participate in the main study (120 per arm) and will be randomly allocated to one of the three study conditions. All participants will be recruited from residential AOD treatment services located in NSW (The Salvation Army, WHOS). Participants attending these programs tend to have had longer term substance use problems (e.g. 19-years [17];) and present with a complex range of co-occurring mental health conditions [13, 14, 17, 18, 20, 21]. Alcohol tends to be the most commonly reported primary substance of dependence across both The Salvation Army and WHOS. This is followed by methamphetamine and opiates [17, 20]. Poly-substance use is common amongst people attending these programs.

### Inclusion / exclusion criteria

Participants will be required to be attending residential services provided by The Salvation Army or WHOS. Participants will be required to [1] have a substance use disorder (i.e. not be attending the service for only a gambling problem), [2] have stayed in the residential facility for at least 4-weeks, and [3] have access to a telephone to complete the intervention when they leave the residential program. Exclusion criteria will be kept to a minimum to promote the generalizability of the results. Participants will only be excluded from the study if they are [1] currently at risk of suicide, [2] have unstable mental health symptoms.

### Recruitment and screening

Continuing care workers (CCWs) will be employed at each of the treatment sites to facilitate the continuing care intervention. CCWs will give presentations at regular intervals to residents at the treatment site that they are employed. These presentations will provide an overview of the Continuing Care Project and will reinforce that participation in the study is voluntary. At the end of these presentations, residents of the treatment facility will indicate whether they are interested in participating in the project using a pen and paper survey form. Only interested residents will be then contacted to discuss participation in more detail with the CCW and review participant information. If the resident decides that they would like to participate at this stage, written consent for participation will be obtained.

The CCW will complete baseline data collection (see Table 1) followed by a Continuing Care plan (see Fig. 1). These will occur face to face with the participant prior to exiting treatment (approximately 1 h session). This will provide an opportunity for the worker to establish rapport with the participants and develop a detailed understanding of the person’s background. When the resident exits treatment, the CCW will make contact via telephone. During this contact, randomisation procedures are completed and contact between the CCW and the participant thereafter is guided by the condition to which the person has been allocated (i.e., 12 x telephone sessions, 4 x telephone sessions or no telephone sessions).

### Randomisation

Eligible consenting participants will be randomly allocated to arms 1, 2 or 3 using central computerized randomization. The allocation sequence will be generated using permuted block randomisation (blocks of size 4) by an independent statistician at the Clinical Research Design, IT and Statistical Support (CReDITSS) unit at the Hunter Medical Research Institute (HMRI). Participants will be stratified based on age (under 25 and over 25) and organisation. This will provide an opportunity to examine the impact of the intervention on young people. At entry, the study participants will be allocated a study number.

### Interventions

Treatment as usual and continuing care plan: Participants allocated to all three arms of the study will continue to complete treatment as usual whilst they are attending the residential treatment program. This will include the standard discharge procedures used at each site (e.g. aftercare plan developed with case manager, encouraged to attend mutual support groups, referrals by the person’s case manager to services in the community). In addition to treatment as usual, all participants will develop a written continuing care plan with the CCW (approximately 1-h session). This plan was taken from the McKay et al. (2010) protocol. It is a take-home resource where participant treatment goals are identified, high-risk situations for relapse are planned for and pro-recovery activities are detailed. This plan involves reviewing the reasons that the participant wants to change their alcohol and/or substance use, identifying and discussing strategies to manage high risk situations, reviewing rewarding activities that the person can engage in once they leave residential treatment, establishing recovering goals, and identifying support people for the participant.

Continuing care interventions: Participants allocated to arms 2 and 3 of the study are offered continuing care telephone sessions in addition to the continuing care plan. The continuing care telephone sessions follow the protocol developed by McKay et al. (2010). The aim is to deliver the sessions weekly (i.e. 12-weeks for Arm 1 and 4-weeks for Arm 2). However, it is recognised that there may be times where sessions are missed, or it is not possible to schedule an appropriate time. Where weekly sessions are missed, participants will be given the opportunity to schedule multiple weekly sessions. For both arms 1 and 2, there is a 12-week period to complete all allocated sessions. The weekly sessions will take between 15 to 30 min to complete. These sessions include a check on mental health symptoms, and counselling around triggers, high-risk situations, coping strategies and recovery-related activities. Participants are encouraged to identify and plan for future high-risk situations and reflect on and set substance-related goals. The procedures for the continuing care telephone intervention also include offering face-to-face appointments or more frequent telephone contact with participants if they are at significant risk of relapse or adverse events [22, 23]. This is in line with recommendations that continuing care interventions offer step-up treatment options for people struggling [4, 6]. Details of any stepped care sessions will be recorded. As the current study is interested in examining the optimal length of continuing care/number of telephone sessions required, the study design includes either a 4-session arm (1-month) or a 12-session arm (3-months).

### Follow-up

Research assistants based at The University of Wollongong will conduct 3- and 6-month post-discharge follow-up assessments over the telephone and will be blind to participant allocation. At the beginning of each phone call, participants will be reminded to not disclose their condition to the research assistant. If participants reveal their condition, an alternative research assistant will complete the assessment. Assessments will take approximately 30 min to complete. Contact (once per day between Monday and Friday, as required) will be attempted by the research assistant rostered that day (telephone call, SMS, email) within 4-weeks of the eligibility date. Alternate contact details provided by the participant will be used if there is difficulty reaching the participant.

### Loss to follow-up

Participants who cannot be contacted within 4-weeks of the 3-month assessment eligibility date will be considered missing at that data collection time point. Research assistants will attempt to contact participants again at the 6-month time point. The exception to this will be if the participant actively withdraws from the study, whereby the participant will be immediately removed from the follow-up calling list.

### Retention

The study will use retention enhancement techniques developed from previous studies [13–15]. These include flexibility in scheduled call times (after hours, weekends) and using text messages to communicate study details with participants or reminders before scheduled calls.

### Reimbursement

Participants will be reimbursed with AUD$40.00 vouchers for baseline assessment, 3- and 6- month assessments. Vouchers will be posted to the current address provided by the participant at each time point.

### Data collection

Data for the project will be collected and initially stored in a Research Electronic Data Capture (REDCap) database, hosted locally on HMRI servers.

### Trial monitoring

#### Intervention delivery

Drug and alcohol workers will be recruited from within The Salvation Army and WHOS to work as CCWs and will be based at treatment sites. Face-to-face training will be completed by the research team (PK, BO, TD) with each of the CCWs. Training will cover research procedures and role-plays of assessment instrument administration, the initial face-to-face session, additional face-to-face sessions if needed, and telephone treatment sessions. Training will be supported by weekly supervision sessions (approximately 1 h a week; led by PK, BO).

#### Treatment fidelity

Prior to commencement of the study the CCWs will be trained to competency in both the assessment and intervention protocols. This will include auditing mock sessions conducted by the clinicians. Once the study commences, all assessment and intervention sessions will be audiotaped. Independent psychologists will rate a random allocation of treatment sessions for fidelity and competence, and provide feedback to the CCWs throughout the study to maintain fidelity. These tapes will also be reviewed within supervision to support adherence.

#### Adverse events

All adverse events will be recorded by CCWs and research assistants between the time of recruitment and the final follow-up assessment.

#### Participant withdrawal

Participants may withdraw at any point during the study without any consequence and this is clearly outlined in the participant information sheet and consent process. No further contact with the participant will be initiated by the research team upon verbal or written withdrawal from study.

### Assessment procedures

#### Primary and secondary outcome measures

##### Primary outcome

The primary outcome will be percentage of days abstinent from alcohol and other drugs (excluding tobacco) over the 28-day period immediately prior to the 6-month follow-up. This will be measured using the well-established Timeline Follow-Back Method [24].

##### Secondary dependent variables

At 3- and 6-months assessment points, secondary analysis will examine self-report recovery outcomes using the Substance Use Recovery Evaluator (SURE), psychological distress using the Kessler-10, confidence using the Drug Taking Confidence Questionnaire (DTCQ-8), Quality of Life using the EUROHIS Quality of Life 8-item index, physical health using Short Form-12, and health literacy using the Health Literacy Questionnaire. The Lifetime Drug Use History questionnaire will be used to assess the range of services that the person accessed following discharge from the residential program (e.g. mutual support groups, general practitioner, other substance abuse treatment). See Table 1 for a list of the measures used in the current study.

File audits of the participants’ electronic records (i.e. The Salvation Army SAMIS system and the WHOS Ted system) will be conducted to identify the care the person received whilst in the facility (i.e. length of stay, attendance at group programs, involvement in mutual support groups, referrals, and engagement in any other forms of care).

### Data analysis

#### Power analysis

A sample of 90 per group will give the study 80% power to detect a 0.3 standardised difference between intervention groups in the change from baseline percentage days abstinent (PDA) at a 5% significance threshold. This calculation assumes a correlation between baseline and follow-up of 0.7. A meta-analysis of continuing care treatment effects for participants with substance dependence disorders identified a pooled treatment standard deviation (SD) of ~ 0.3SD (reference?). A SD of 30% for PDA has been reported elsewhere [24], so a 0.3 SD difference corresponds to a clinically meaningful difference of 10% between the treatment groups [25]. Based on a follow-up of 75% of participants at 6-months, we will recruit 360 participants to the study to ensure sufficient power.

#### Analysis plan

The analysis will follow the intention to treat principle. The difference between treatment groups in the primary outcome will be assessed using a linear regression model. The outcome in the model will be percentage days abstinent at 6-month follow-up, and the model will include fixed effects for the baseline value of the outcome, treatment group, and the stratification variables. Significance tests of the differences between treatment groups at 6-months will be based off estimated marginal means using a Wald-based *t*-test. Modelling assumptions will be assessed using graphical techniques, and appropriate changes made if the assumptions are violated (bootstrapping standard errors for example). Differences in secondary outcomes will be assessed using similar models for continuous outcomes; and logistic regression models for dichotomous outcomes. Generalised linear mixed models will be used for comparison of outcomes that are measured at multiple post-baseline time-points using an unstructured residual correlation matrix and fixed effects for time, treatment and the interaction between time and treatment. Missing data will be imputed multiple times using the chained regression equations method, with treatment effect estimated pooled across imputations using Rubin’s method.

#### Economic evaluation

Taking a health sector perspective, a within-trial cost-effectiveness analysis will be based on the relative change, between baseline and 6-month post intervention, in the primary outcome variable: percentage days abstinent. This will be done comparing the three trial arms: usual continuing care versus 4 sessions, usual continuing care versus 12 sessions, and 4 sessions versus 12 sessions. Cost collection will include three elements: the cost of delivering the sessions (e.g. staff time, training and supervision), the flow-on cost impacts to other services (e.g. allied health and hospitalisations) and client out-of-pocket costs (e.g. co-payments) using a modified Client Services Receipt Inventory (CSRI; 26) (CSRI). In addition, the economic evaluation will be repeated in a cost-utility analysis where the measure of outcome is health-utility which is an economic measure of health-related quality of life derived from the SF-12. A full uncertainty analysis will be undertaken including an assessment of the value of undertaking further research.

## Discussion

The purpose of the Continuing Care Project is to trial a continuing care telephone intervention for people leaving residential AOD treatment in Australia. Strengths of the study include participant recruitment from multiple service providers (i.e., The Salvation Army, WHOS) across four treatment sites. Treatment sites from urban and regional areas of New South Wales have been included in an effort to increase the heterogeneity of the sample and enhance the generalizability of the findings. Exclusion criteria for the study has also been kept to a minimum to promote generalizability. For example, the study includes both males and females, participants with with co-occurring mental illness and has no exclusionary criteria based on primary substance. The proposed trial will extend on the existing research on the continuing care intervention [10–12] by recruiting a sample from residential treatment services rather than from outpatient services. Providing opportunity to examine whether the clinical and cost effectiveness of the intervention found in outpatient samples is applicable to those receiving inpatient treatment from residential settings.

Challenges of completing intervention research at residential substance use services has been documented [15]. These challenges include the high rates of unplanned attrition and difficulty retaining participants for follow-up assessment. Tending too these challenges, continuing care plans will be completed within seven days of the baseline assessment. Since participants will only be eligible for randomization after the continuing care plan is complete, this strategy will help retain participants to the point of treatment allocation. Attempts to improve follow-up rates in the current study will include using telephone follow-up, obtaining contact details of significant others to help with locating participants and financially compensating participants for the time required to complete the assessments (AUD$40.00).

## Conclusion

The proposed study addresses calls in the broader academic literature to conduct well-controlled studies of continuing care interventions for residential substance use populations [4–6]. The study will provide important information on the clinical effectiveness and cost effectiveness of including continuing care telephone interventions as part of AOD services. It is anticipated that the current study will demonstrate that a continuing care intervention is a relatively low-cost clinical intervention that can help to support people following residential care. The study will also provide evidence on the number of telephone sessions required to improve abstinence and other outcomes as part of the continuing care intervention. Results from the current study may also help to inform the implementation of continuing care interventions for other outcomes that can affect people accessing AOD treatment (i.e. mental illness, poverty, homelessness, criminal involvement).

## Trial status

The trial is registered with the Australian New Zealand Clinical Trials Registry - ACTRN12618001231235. It was first registered on 23rd July 2018 and the most recent update to the trial was 25th June 2019. To date, 216 participants have been randomized. Recruitment of participants is expected to be completed by December 2019.

## Supplementary information

**Additional file 1.** SPIRIT Checklist.

## Data Availability

Not applicable.

## Abbreviations

AOD: Alcohol and other drug
CCW: Continuing care worker
HMRI: Hunter Medical Research Institute
PDA: Percentage of days abstinent
WHOS: We Help Ourselves
