# Correction to: Study protocol the Continuing Care Project: a randomised controlled trial of a continuing care telephone intervention following residential substance dependence treatment

**DOI:** 10.1186/s12889-020-8328-2

**Published:** 2020-02-19

**Authors:** Peter Kelly, Frank Deane, Amanda Baker, Gerard Byrne, Tayla Degan, Briony Osborne, Camilla Townsend, James McKay, Laura Robinson, Christopher Oldmeadow, Kenny Lawson, Andrew Searles, Joanne Lunn

**Affiliations:** 1 School of Psychology, University of Wollongong, Northfields Avenue, Wollongong, New South Wales 2522 Australia; 2 Illawarra Health and Medical Research Institute, University of Wollongong, Northfields Avenue, Wollongong, New South Wales 2522 Australia; 3 University of Newcastle, University Drive, School of Medicine and Public Health, Callaghan, New South Wales 2308 Australia; 4 The Salvation Army, Chalmers Street, Redfern, New South Wales 2016 Australia; 5 University of Pennsylvania, Market Street, Philadelphia, PA 19104 USA; 6 Hunter Medical Research Institute, Kookaburra Circuit, New Lambton Heights, New South, Wales 2305 Australia; 7 We Help Ourselves, Rozelle, New South Wales 2039 Australia

**Correction to: BMC Public Health**

**https://doi.org/10.1186/s12889-020-8206-y**

It was highlighted that in the original article [1] author Amanda Baker was erroneously omitted from the authorship during the copy editing stage. This Correction article shows the correct authorship. The original article has been updated.
