# Supplementary material for: Study protocol the Continuing Care Project: a randomised controlled trial of a continuing care telephone intervention following residential substance dependence treatment
Source: BMC Public Health. 2020 Jan 28;20:107. doi: 10.1186/s12889-020-8206-y (PMC6986107; doi:10.1186/s12889-020-8206-y)
Supplement: Supplementary file 1 — Additional file 1. SPIRIT Checklist. [file 12889_2020_8206_MOESM1_ESM.pdf]

NSW GOVERNMENT | Health

H17/51861-5

gerard.byrne@aue.salvationarmy.org

Dear Mr Byrne

**Re: AOD Innovation Grants Scheme Round 1 outcomes**

I am delighted to advise that your application to Round 1 of the AOD Innovation Grants Scheme for the project 'A randomised controlled trial of a continuing care telephone intervention following residential substance abuse treatment' has been successful.

Your proposal was one of five full applications received in the current round. All full applications were assessed through a competitive process by the AOD Innovation Grants Scheme Independent Selection Panel, with advice from other relevant experts where required.

The Selection Panel has requested some adjustments to your proposal, outlined in the attached document. These requests are made with the intention of ensuring the best possible outcome for your project. The changes will have an impact on your proposed budget. Please review the feedback, incorporate all changes in track on your original full application and return a pdf and word version to aodgrants@moh.health.nsw.gov.au as soon as possible.

A Selection Panel member will be required to review and endorse the revised application. Once endorsed, Genevieve Whitlam, Senior Program Manager, will contact you regarding the final funding amount and your Funding Agreement. The Ministry will need to be notified of progress of the research project and financial acquittal of grant funds, including notification of any unspent funds. Reporting requirements for the grant are outlined in the Funding Agreement. Please also notify the Ministry if you receive other sources of funding for this research proposal.

I urge you to start any processes for recruitment of project staff, and ethics and governance approval as soon as you are advised that your revised application has been endorsed by a Selection Panel member. Given the relatively short timeframes of the grant period, starting these processes as soon as possible will have a significant impact on project timelines.

NSW Health will be announcing the successful applicants shortly. Until this announcement please do not initiate local media in regard to the success of your proposal.

For any queries or feedback please email aodgrants@moh.health.nsw.gov.au.

Congratulations on the success of your full application. Thank you for your commitment to research that will contribute to the health and well-being of people in NSW. I look forward to updates on the progress of your research project.

Yours sincerely

Dr Kerry Chant PSM
**Chief Health Officer and Deputy Secretary**
**Population and Public Health**

14/8/17.

**NSW Ministry of Health**
ABN 92 697 899 630

73 Miller St North Sydney NSW 2060
Locked Mail Bag 961 North Sydney NSW 2059
Tel. (02) 9391 9000 Fax. (02) 9391 9101
Website. www.health.nsw.gov.au

**AOD Innovation Grants Scheme Full Application: Selection panel comments**

| | |
|---|---|
| Application Number: | 206 |
| Chief Investigator: | Gerard Byrne |
| Host Organisation: | The Salvation Army (NSW) Property Trust |
| Project Title: | A randomised controlled trial of a continuing care telephone intervention following residential substance abuse treatment |
| Budget requested: | $727, 225 |

The following feedback from the AOD Innovation Grants Scheme Independent Selection Panel should be incorporated into the final research project proposal before it commences. Milestone reports for the funds at the end of year one and two will need to demonstrate that these comments have been addressed.

- The Selection Panel views your project as a good opportunity to test a systematic approach to telephone follow-up. However, they request that the study design be amended to understand more about the effective dose of the intervention. Please amend the design from a 2-arm randomised controlled trial (RCT) to a 3-arm RCT, with the following arms:
  - Usual care only
  - Usual care plus 4 telephone calls (new arm)
  - Usual care plus 12 telephone calls
- The Selection Panel has questions about the need and scalability of employing 3 site-specific telephone after-care support workers. It is requested that the proposal change to include a more centralised approach to the phone support service for this trial (e.g. one or two people from one call-centre location for the trial). This increases the potential for scalability of the intervention and ensures a more systematic approach across all follow-up phone calls.
- The Panel highly recommends the collection of implementation data, particularly about "usual care" (e.g. what did they receive as usual care – referral, aftercare, 12-step program).
- Please include your economic analysis, as designed.
- The changes requested above will have an impact on your proposed budget. Please revise accordingly.
